# Imaging of Spondylodiscitis: A Comprehensive Updated Review—Multimodality Imaging Findings, Differential Diagnosis, and Specific Microorganisms Detection

**DOI:** 10.3390/microorganisms12050893

**Published:** 2024-04-29

**Authors:** Amandine Crombé, David Fadli, Roberta Clinca, Giorgio Reverchon, Luca Cevolani, Marco Girolami, Olivier Hauger, George R. Matcuk, Paolo Spinnato

**Affiliations:** 1 Department of Musculoskeletal Imaging, Pellegrin University Hospital, Bordeaux University, Place Amélie Raba-Léon, F-33000 Bordeaux, France; 2 Department of Radiology, IRCCS Policlinico di Sant’Orsola, 40138 Bologna, Italy; 3 Diagnostic and Interventional Radiology, IRCCS Istituto Ortopedico Rizzoli, 40136 Bologna, Italy; 4 Orthopedic Oncology Unit, IRCCS Istituto Ortopedico Rizzoli, 40136 Bologna, Italy; 5 Department of Spine Surgery Unit, IRCCS Istituto Ortopedico Rizzoli, 40136 Bologna, Italy; 6 Department of Imaging, Cedars-Sinai Medical Center, Los Angeles, CA 90048, USA

**Keywords:** magnetic resonance imaging, multidetector computed tomography, image-guided biopsy, positron emission tomography computed tomography, spondylodiscitis

## Abstract

Spondylodiscitis is defined by infectious conditions involving the vertebral column. The incidence of the disease has constantly increased over the last decades. Imaging plays a key role in each phase of the disease. Indeed, radiological tools are fundamental in (i) the initial diagnostic recognition of spondylodiscitis, (ii) the differentiation against inflammatory, degenerative, or calcific etiologies, (iii) the disease staging, as well as (iv) to provide clues to orient towards the microorganisms involved. This latter aim can be achieved with a mini-invasive procedure (e.g., CT-guided biopsy) or can be non-invasively supposed by the analysis of the CT, positron emission tomography (PET) CT, or MRI features displayed. Hence, this comprehensive review aims to summarize all the multimodality imaging features of spondylodiscitis. This, with the goal of serving as a reference for Physicians (infectious disease specialists, spine surgeons, radiologists) involved in the care of these patients. Nonetheless, this review article may offer starting points for future research articles.

## 1. Introduction

### 1.1. Epidemiology

Spondylodiscitis is an infection involving the intervertebral discs and/or adjacent vertebral bodies. Most cases are due to bacterial infection (pyogenic), but tuberculous and fungal etiologies can also occur, especially in immunocompromised patients. Spondylodiscitis represents 2–5% of all cases of osteomyelitis [1]. In Europe, the incidence ranges from 4 to 24 per million per year [2,3]. Although infection is more common in older patients, there is a bimodal distribution with peaks less than 20 years and between 50–70 years, with spondylodiscitis comprising 1–2% of pediatric bone infections [4]. There is a 1.5–2:1 male-to-female predominance, particularly in older populations, probably due to a higher frequency of comorbidities in men over 60 years old [4]. 

There has been an increasing incidence of spondylodiscitis in recent years due to increasing numbers of older patients with chronic diseases such as diabetes mellitus and renal failure, steroid and other immunosuppressive therapies, sickle cell disease, Human Immunodeficiency Virus (HIV) infection and other immunocompromised states, and intravenous drug abuse [5,6]. Direct inoculation may follow spinal surgery and procedures with a prevalence up to 18.8% [7]. In South Korea, the incidence rate of pyogenic spondylodiscitis per 100,000 people increased from 15.35 in 2010 to 33.75 in 2019, although tuberculous spondylodiscitis decreased from 7.55 in 2010 to 2.04 in 2019. In Germany, there was a 41.6% rise in cases between 2010–2020 to 14.4/100,000 inhabitants, with 59.6% cases in patients 70 years or older and 56.2% involving the lumbar spine [8]. In Germany, inpatient mortality from pyogenic spondylodiscitis also rose by 347% from 2005–2021 [9]. 

Most cases of spondylodiscitis are a result of hematogenous spread, as may occur with bacteremia [4]. One-third of patients with pyogenic spondylitis have endocarditis, with 2–20% of patients with endocarditis developing spondylodiscitis [10]. Spondylodisitis and infected endocarditis are frequently associated. Therefore, especially if they have risk characteristics, patients with infectious endocarditis should be evaluated for “metastatic” infection of the spinal column. On the other hand, neither the short-term nor long-term prognosis for individuals with infected endocarditis appears to be deteriorated by spondylodiscitis.

In adults, the intervertebral discs are avascular, so septic emboli produce ischemia and infarction of the vertebral endplates (especially at their anterior side) with subsequent bone destruction and disc involvement [4]. In contrast, in children, intra-discal anastomoses remain open, and infection can be limited to the disc. Although arterial spread is more common, retrograde venous spread can also occur with pelvic organ or retroperitoneal infection. Direct inoculation secondary to spinal surgery or procedures accounts for approximately 20% of cases [11].

Staphylococcus species (most commonly *S. aureus*) is the most frequent causative organism, accounting for approximately 40–67% of cases [12]. Although most are susceptible to methicillin, methicillin-resistant *Staphylococcus aureus* (MRSA) strains are becoming more frequent [4]. The most common gram-negative organisms are *Escherichia coli*, Pseudomonas, and Proteus species [13]. In the setting of infectious endocarditis, there is an over-representation of *Streptococcus*, whereas sickle cell disease predisposes to Salmonella. Approximately 30% of cases are caused by *Mycobacterium tuberculosis*, and most other cases are caused by other bacteria [12]. Only 0.5% of cases are fungal in etiology, and viral and parasitic infections are rare. However, the vertebra is the most common site of fungal osteomyelitis, with about one-third of fungal infection of the spine due to *Aspergillus* and another third due to *Candida* [14,15]. No causative organism is isolated in 21–34% of cases [16].

**Figure 1:**
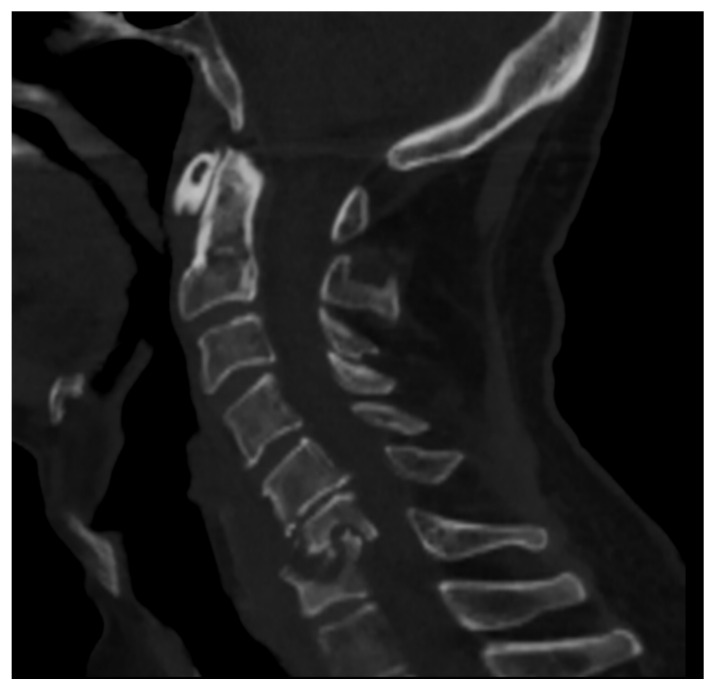
CT of the cervical spine (sagittal reconstruction) of a middle-aged man (HIV+) affected by spondylodiscitis in the C6–C7 tract of the cervical spine complicated by myelopathy.

The lumbosacral region is involved in 52–58% of cases, the thoracic spine in 26–35% of cases, and the cervical spine in the remaining 10–22% of cases (Figure 1) [12]. 

Single-level involvement occurs in 65% of patients, against 35% of patients with multiple-level involvements (including 10% with no contiguous levels). The median delay to diagnosis for pyogenic infection is 30 days, with microbiological diagnosis established in approximately three-quarters of cases [17]. The median duration of antibiotic therapy is 148 days [16,17]. Although conservative treatment can be effective for 70% of cases of pyogenic infection, surgery may be required in over 50% of cases for all causes [12,17,18]. For pyogenic infection, complete healing without disability occurs in over three-quarters of cases; there is an overall healing rate of 91%, with 24% residual disabilities and a mortality rate of 8% [17]. Adverse prognostic factors include negative microbiological culture, neurological impairment at diagnosis, and endocarditis [17].

### 1.2. Complications

Complications include neurological compromise, abscess, and instability in 27.8%, 30.4%, and 6.6% of cases, respectively [12]. Most abscesses (60%) occur in the lumbosacral region, with 33% in the thoracic region and 7% in the cervical region [12]. Epidural abscesses are most common in the cervical region with spinal cord compression and neurological compromise in up to 56% and 65% of cases in this region [12]. Epidural abscesses and neurological compromise require surgery in 86% and 84% of cases, respectively [12]. Most (95%) of paravertebral abscesses can be treated percutaneously [12]. Most (53%) of cases of instability involved the lumbosacral region, with surgery required in 87% of cases of instability [12]. Despite improved antibiotic and surgical treatments, patient hospital stays can average 30–57 days, and mortality can be as high as 2–17% [18,19,20,21].

### 1.3. Clinical Features

The clinical diagnosis of spondylodiscitis can be challenging, with some patients presenting with non-specific symptoms that can overlap with or be obscured by other coexisting conditions, such as spondylosis, prior spinal surgery, cardiovascular conditions, and diabetes mellitus [22,23,24]. Most patients (93%) present with stabbing, intense back pain that can radiate to the limbs and worsen at night or with weight-bearing [1,4,23]. Pyogenic spondylitis can be painless in 7% of patients, with these patients more likely to be intravenous drug abusers or have liver failure/cirrhosis, higher rate of surgery (38% vs. 16%), more commonly infected with *E. coli* or *Pseudomonas* spp., and with double the mortality [23].

Many patients report a febrile illness in the weeks before the onset of back pain with a long period of defervescence [4]. Only half of patients present with fever with paraspinal muscle tenderness present in 75–95% [23]. Neurological deficits are the presenting symptom in up to a third of cases, ranging from abnormal sensation, radiculopathy, weakness, and even severe paralysis and bowel and bladder incontinence [10,25]. Patients may present with toxic infectious symptoms such as anorexia, nausea, and vomiting in 5 to 50% of cases [17,26]. Cases with a long delay in diagnosis may present with weight loss [4].

Compared to pyogenic spondylitis, tuberculous spondylitis is more commonly associated with younger age, longer duration of symptoms, absence of fever, thoracic spine involvement, greater than three levels of vertebral involvement, and presence of a paraspinal abscess [27]. 

Clinical predictors for fungal discitis/osteomyelitis include back pain for 10 or more weeks, antibiotic use for 1 week or more, and intravenous drug abuse [28].

### 1.4. General Biological Features

Erythrocyte sedimentation rate (ESR) and C-reactive protein (CRP) levels are almost always elevated [4]. CRP is more sensitive as a marker for treatment response, with a drop of 50% per week, and is a good predictor for disease treatment [20]. White blood cell (WBC) count may be elevated or within the normal range and thus is not particularly useful for making the diagnosis [29]. Blood cultures are positive in 30–78% of cases of pyogenic spondylodiscitis [25]. In cases with negative blood cultures, discal or vertebral percutaneous CT-guided needle or surgical biopsy may be indicated, with diagnostic yield rates ranging from 47–100% [25]. It must be noted that these biological features can be missing with tubercular spondylodiscitis and intracellular development microorganisms (such as *Brucella* spp., *Legionella* spp., and *Listeria* spp.).

## 2. Imaging in the Initial Assessment of Suspected Spondylodiscitis

Imaging plays a fundamental role in the early assessment of suspected spondylodiscitis. An early imaging diagnosis is usually considered the detection of the disease when only minor vertebral and disk damages are present. Moreover, it also refers to a diagnosis performed closely to the symptom’s onset. The main tools for early (<2–3 weeks after the onset of symptoms) and later diagnoses are the following.

### 2.1. Conventional Radiographs

Radiographs exhibit low sensitivity and specificity, making them less effective in detecting bone loss. Changes in pyogenic spondylitis are not apparent on plain radiographs until 2–8 weeks after the initial symptoms. They may, in fact, remain normal for several weeks after infection. To identify bone loss, a substantial 30% to 40% reduction in the bone matrix is necessary, a process that may extend beyond 2 weeks, especially during acute infections [6,30,31,32]. 

**Figure 2:**
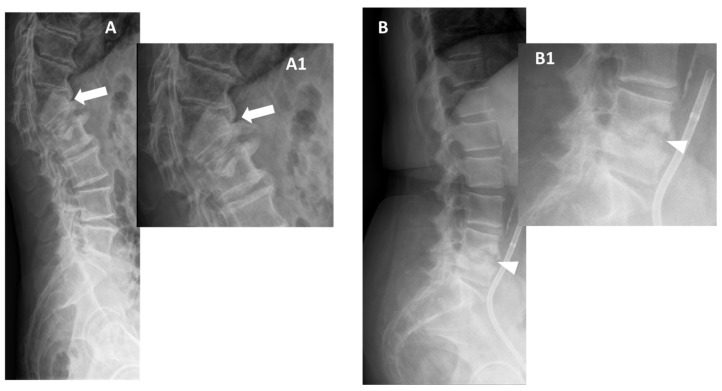
Conventional radiographs, lateral views (Panel **A**, and Magnification **A1**), of a 77-year-old male with previous pyogenic spondylodiscitis of T12-L1 vertebral bodies (partially collapsed and fused—arrows). Conventional radiographs, and lateral views (Panel **B** and Magnification **B1**) of a 64-year-old female with spondylodiscitis of L4–L5 vertebral bodies (thick endplate erosions are detected—arrowheads).

In the early stages of infection, specific radiographic markers are rarely evident, making it challenging to differentiate from degenerative pathologies. After 8 to 12 weeks, obvious destruction of bones can be observed (Figure 2) [32].

As the disease advances, observable changes include nonspecific osteopenic changes (demineralization) in the subchondral layer, erosive and blurred endplate margins, diminished intervertebral space, paravertebral soft tissue mass, resultant deformities, and noticeable soft tissue swelling.

End plate erosion is often subtle but recognized as the most reliable sign that can be detected on plain films and is the single most important observation to be made in evaluating any radiograph of the lumbar spine [30]. In cases of chronic infection, spinal deformities like kyphosis, scoliosis, or a combination of both may become apparent after approximately four months.

In the setting of spinal tuberculosis in the upper lumbar and lower thoracic spine, radiographs serve as the primary diagnostic tool, demonstrating 82% sensitivity, 57% specificity, and 73% accuracy [33]. Characteristic radiographic findings include rarefaction of vertebral end plates, disk height loss, osseous destruction, new bone formation, and soft tissue abscess, often leading to gibbus deformity and vertebral collapse. Additionally, concomitant pulmonary tuberculosis is common, with up to 67% of patients having associated primary lung focus or a history of pulmonary tuberculosis [34].

### 2.2. Computer Tomography (CT)

CT stands out in its ability to detect bone changes earlier than radiographs. Although it can also be normal within the first three weeks, it can later reveal:

- Fragmentation or erosive changes in the vertebral endplates;
- Ill-defined reactive sclerosis or osteopenia;
- Intra-discal hypodensity;
- Soft tissue swelling that obscures the fat planes surrounding the vertebral body.

The use of intravenous contrast can enhance the visibility of the epidural venous plexus, aiding in the assessment of the extent of the mass effect on the thecal sac [34]. It also allows for the identification of surrounding swellings and thickenings in the paravertebral fat tissue, heightened enhancement, abscess formation (often in the psoas muscle or in the epidural space), and the recognition of gas inclusions suggestive of inflammatory soft tissue infection—even though this can be seen with degenerative discitis [35].

**Figure 3:**
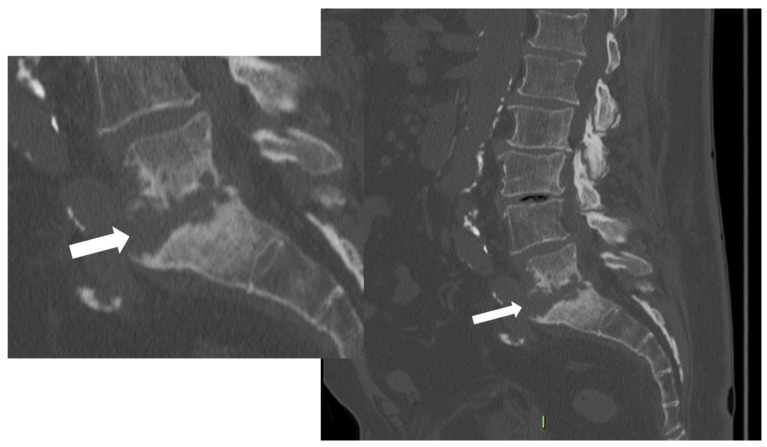
CT, sagittal reconstruction (magnification on the left), of a 77-year-old female with L5-S1 pyogenic spondylodiscitis characterized by thick endplates erosions (arrows).

As the infection progresses, CT may show soft tissue replacement of the bone. The involvement of the bone can result in erosive changes to the end plates (Figure 3).

Additionally, direct inoculation of the disk space might occur, involving the subjacent end plate and potentially leading to the collapse of the disk space [36,37].

Additionally, if an abscess is present, CT-assisted punctures can be performed to obtain tissue samples for microbiological diagnostics. Furthermore, CT is often recommended as the initial method for puncturing suspicious tissue (intervertebral disk, vertebral body) to identify causative germs (see Section 3.2 focused on image-guided biopsy). CT can also be particularly useful in patients for whom MRI is contraindicated because of implanted devices or if MRI is unavailable.

### 2.3. MRI

Contrast-enhanced MRI stands out as the preferred imaging method for diagnosing spinal infections, having a sensitivity of 97%, specificity of 93%, and an accuracy of 94% for diagnosing spondylodiscitis [37,38,39,40,41]. It excels in revealing the infection’s extent, providing superior images of paraspinal soft tissues and the epidural space. However, it could appear normal within the first 2–4 days [37,38,39,40,41].

MRI protocols suggest utilizing fat-suppressed T2-weighted imaging (WI) sequences and post-gadolinium T1-WI with fat suppression in the study of these conditions [42]. Alternatively, the DIXON T2-WI and contrast-enhanced (CE) T1-WI sequences can be used (with the Fat, Water, and In phase images).

Although not routinely used, diffusion-weighted imaging (DWI) in patients who cannot undergo contrast-enhanced MRI due to contraindications, such as allergic reactions and renal impairment, could help in the detection of abscesses, and provides some additional clues to guide diagnosis. DWI is also useful in differentiating infection from degenerative changes and distinguishing normal postsurgical fluid collections from infected ones. The use of DWI, however, is still debated because of its moderate-to-low sensitivity in differentiation between spondylodiscitis and other differential diagnoses. 

**Figure 4:**
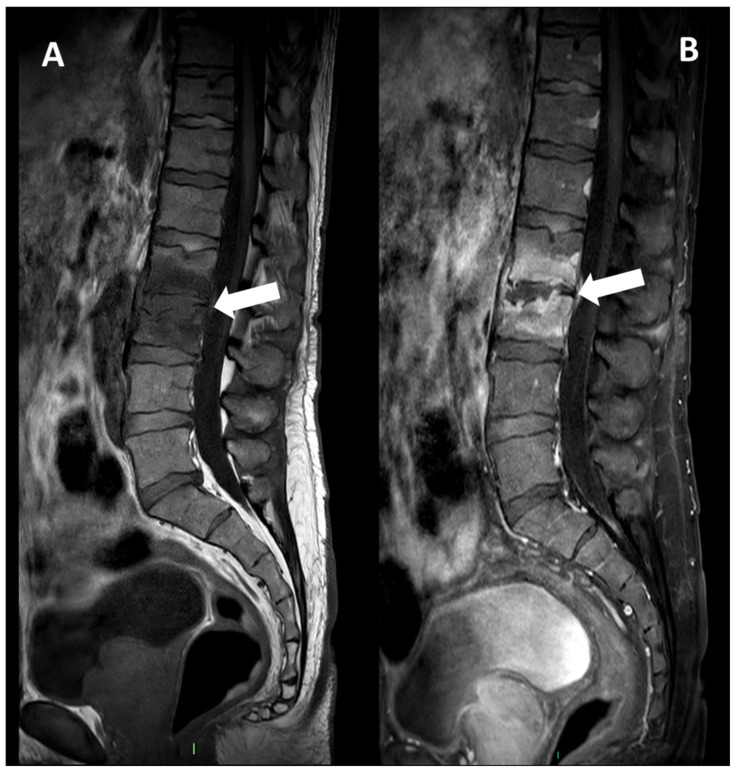
MRI, Sagittal T1w (Panel **A**), and T1w after contrast media injection (Panel **B**) of a 54-year-old male with pyogenic spondylodiscitis of L2-L3 vertebrae: complete alteration of disc signal intensity, endplates erosions, and diffuse pattern of vertebral body enhancement are detectable (arrows).

Spondylodiscitis induces inflammatory exudate that replaces normal marrow with white cells and causes hyperemia. This leads to changes in MRI signals, manifesting as hypo- or isointense T1 and hyperintense T2 signal intensities in the subchondral end plates and intervening disc. Typically, signal alterations initiate in the anterior aspect of the vertebral body, affecting single or multiple spinal segments. It can be unilateral at the early stage of the disease. Again, bone erosions of the endplates are observed. The contrast enhancement of the vertebral endplate can demonstrate various patterns, namely diffuse, patchy, clumped, or linear enhancement parallel to the endplate (Figure 4). 

This process results in the loss of end plate definition, diminished disc height, potential presence of a positive nuclear-cleft sign, and, in later stages, vertebral body destruction. 

The nuclear cleft, a band with low signal intensity on T2-WI in the noninfected disc, becomes distorted and then effaced in the presence of infection; however, this sign is not specific to spondylodiscitis and can also occur in degenerative disc disease. Later, a high signal intensity on T2-WI can be visible, which, later can enhance after gadolinium chelates injection. Afterward, the disk height decreases.

After gadolinium contrast administration, enhancement of the subchondral bone or vertebral body may be observed, and the affected disc shows diffuse enhancement. In some cases, the presence of an abscess with T1 hypointensity, T2 hyperintense, and contrast enhancement within the disc or bone can be identified. 

In a second time, the infection can spread to the epidural space and the paravertebral soft tissues, which translates to imaging in a phlegmonous ill-defined infiltrate (with high signal intensity on fat-suppressed CE-T1-WI and T2-WI) and paraspinal abscesses (with pyogenic centrum displaying low signal intensity on T1-WI, high fluid-like signal intensity on T2-WI without contrast uptake, a peripheral rim showing contrast-enhancement on fat-suppressed CE-T1-WI).

Notably, pyogenic spondylodiscitis generally less frequently affects the posterior elements of the spine. Key findings with high sensitivity for diagnosing pyogenic spondylodiscitis include paraspinal or epidural inflammation, vertebral body T1 hypointensity, disc space T2 hyperintensity, and disc space enhancement [43]. 

Furthermore, MRI findings could aid in distinguishing spinal tuberculosis from pyogenic spondylodiscitis (see Section 3.1 focused on MRI on differential diagnosis between microorganisms involved) [44]. Some of them include the presence of a large, well-defined paraspinal abscess with thin rim enhancement and smooth margins, involvement of the thoracic spine, subligamentous extension to adjacent vertebrae with preserved disc height, and multilevel involvement with skip lesions [43]. In cases of neurological deficit, an MRI is crucial for planning surgical approaches and determining the levels of decompression and stabilization. If available, a comprehensive spine MRI is optimal for assessing skip abscesses and other areas of neurologic compression.

### 2.4. Nuclear Medicine

To enhance the diagnosis of unclear radiologic findings in suspected spinal infections, radionuclide imaging procedures, such as technetium-99m scintigraphy and gallium-67 scintigraphy, can be employed with varying sensitivities and specificities. 

Combining three-phase technetium-99m scintigraphy with CT or other techniques enhances diagnostic accuracy by localizing infections and excluding differential diagnoses. This modality is highly sensitive, but non-specific.

**Figure 5:**
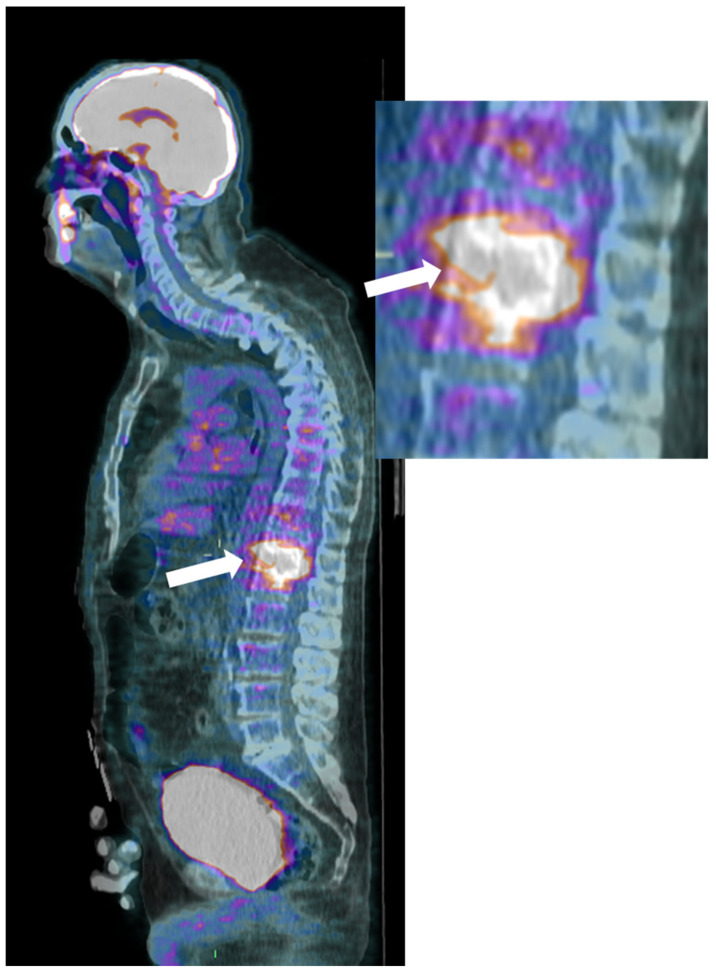
(FDG) PET-CT, Sagittal reconstruction (magnification on the right) of a 72-year-old male with pyogenic spondylodiscitis of T12-L1 vertebrae. A high pathologic FDG uptake is detected in the disc and vertebral endplates (arrows).

Finally, ^18^F-Fluorodeoxyglucose-positron emission tomography (^18^F-FDG-PET) is a sensitive and whole-body imaging tool, though lacking anatomical details; combining it with CT or MRI improves spatial resolution and aids in distinguishing infectious from degenerative abnormalities (Figure 5) [42,45]. 

It can be interesting to detect multiple intra- and extra-osseous infectious locations with high sensitivity but low specificity, with standardized uptake values (SUV) between 4 and 30 [46]. If required, ^18^F-FDG-PET/CT can help monitor the treatment efficacy, with a 39% decrease being found in responding patients on ^18^F-FDG-PET/CT performed at 2 weeks after starting antibiotherapy [44,47]. Additionally, a SUV_max_ decrease above 15% at 2 weeks would indicate a good treatment response with a sensitivity of 94% and specificity of 67%, i.e., with higher accuracy than concomitant conventional MRI (sensitivity = 37% and specificity = 50%) [44]. However, it must be remembered that there is no routine indication for repeating MRI or 18F-FDG-PET/CT to evaluate the response to treatment.

In a retrospective analysis by Love et al. [48], bone scintigraphy conducted on 22 patients with suspected spondylodiscitis (SD) showed planar imaging to have 73% sensitivity, 31% specificity, and 50% overall accuracy for detecting infection. Sensitivity increased to 82%, while specificity decreased to 23% when single-photon emission computed tomography (SPECT) was utilized, maintaining an overall accuracy of 50%. When SPECT images were independently interpreted, the test demonstrated 73% sensitivity, 69% specificity, and an accuracy of 71%. 

Despite their efficacy, these techniques are costly and limited in availability, and currently, the Infectious Diseases Society of America (IDSA) guidelines recommend the use of ^18^F-FDG-PET/CT only in cases where an MRI is contraindicated [42,45].

In Table 1 we summarized the main diagnostic characteristics of imaging tools for the diagnosis of spondylodiscitis.

### 2.5. Imaging of Associated Conditions

Spondylitis without discitis. Isolated spondylitis without discitis can be encountered either at the very early stage of a classical infectious spondylodiscitis or in old or immuno-compromised patients. CE MRI is the best imaging modality as it clearly demonstrates bone edema with a contrast enhancement on fat-suppressed CE-T1-WI, associated with a paraspinal inflammatory infiltrate. Later, intra-vertebral abscess and pathological bone fracture and collapse can occur. In the setting of tubercular spondylitis, bone sclerosis can be seen (providing an ivory vertebra, generally at multiple levels) due to the reactive inflammation surrounding the infection [49].

Facet joint infections. Zygapophyseal joints are the sole synovial joints of the spine. They can be infected either hematogenously, through direct inoculation, or via the diffusion of an epiduritis via the retrodural space [50]. The facet joint infections are mostly due to *S. aureus* and are likely to be underdiagnosed [51]. They can be single or multiple levels, uni- or bilateral, and associated with infectious spondylitis. The best imaging modality is CE MRI, which can demonstrate non-specific findings such as fluid in the joint capsule with a contrast enhancement of the synovial, bone marrow edema of the subchondral bone of the facet, bony erosions and edema, pyomyositis, and abscesses in the adjacent soft tissues [51].

## 3. Specific Microorganism Diagnosis 

Imaging can serve as a diagnostic tool in the detection of the possible microorganisms involved in the infectious process. The microorganism identification can be supposed by the analysis of imaging features displayed (MRI above-all) or can be reached by the mini-invasive image-guided biopsy procedures (CT-guided above-all).

### 3.1. MR and Other Imaging Tools in the Differential Diagnosis between Tubercular and Pyogenic Spondylodiscitis

MRI is considered the gold standard for the diagnosis of spinal infections, with a sensitivity of about 96%, specificity of 93%, and accuracy of 94%. The study’s accuracy is significantly increased by contrast enhancement infusion with gadolinium [52]. MRI is crucial, especially when the isolation of the microorganism involved is not achievable and could help in choosing the correct targeted antibiotic therapy thus avoiding complications such as abscess formation, spinal deformities, and neurological deficits.

Many studies since the early 2000s have investigated the differences between the two most common types of spondylodiscitis, pyogenic (PyS) and tubercular (TbS), identifying some specific MRI features for differential diagnosis [52,53,54,55,56,57,58,59,60,61,62,63,64,65,66].

Two features are especially correlated with TbS in almost all studies, namely thoracic involvement and the presence of more than two vertebral elements affected with multiple and non-adjacent vertebral bodies involved (skip lesions). Usually, the infection starts in the anterior subchondral region of the vertebral body and spreads frequently to the anterior longitudinal ligament and other subligamentous areas [53]. Involvement of posterior elements is also more common in this type of spondylodiscitis, even if the vertebral bodies are more frequently affected than the posterior arches [49,50,56]. Especially when there is relative disc preservation, posterior lesions need to be differentiated from neoplastic ones. In this setting, it may be useful considering that tubercular infections classically spread to soft tissue and adjacent ligaments in an anterolateral direction [50].

Virtually all studies have found well-defined paraspinal abnormal signal intensity with intraosseous, epidural, and paraspinal abscesses more frequently in TbS [49,50,51,52,53,54,55,56]. Thin and smooth enhancement of the abscess wall is one of the most reliable MRI findings of TbS (with possible calcifications), whereas ill-defined paraspinal abnormal signal and thick and irregular enhancement of the wall abscess are suggestive of PyS [50,51,52,53]. The chronic course, the relative late phase of TbS and the very minimal inflammation of these types of abscesses (named ‘cold abscesses’) are probably associated with this typical appearance of the abscess wall. Thus, contrast-enhanced infusion is necessary to differentiate these two kinds of spondylodiscitis [51,52,53,54]. 

The size of the paravertebral abscesses is also usually significantly larger in TbS than in PyS and they are often symmetrical. A psoas abscess was found to be a typical feature of this type of spinal infection [53]. The epidural abscess is also significantly more common in TbS and associated with a higher frequency of nerve and spinal cord compression [54,55,56]. Frel et al. showed that meningeal enhancement at the level of the pathological spinal segment was strongly associated with TbS [57,58].

Patients with PyS have limited vertebral injury, and most pathologic alterations are limited to the end plate. On the other hand, in TbS, more than half of vertebral bodies are involved, and they are frequently severely damaged. Vertebral loss of height and collapse with kyphosis (with possible spinal cold injury) most frequently occur in TbS in the thoracic spine and are generally seen in the later stages of tuberculosis [53,55]. Thus, large geodes, bone scalloping, sequestrum, vertebral fragmentation, and ivory vertebra (due to sclerosing response to osteonecrosis) are more typical of TbS compared to PyS.

In TbS the narrowing of the disc space occurs later and is not as pronounced as in PyS. The relative preservation of the intervertebral disc is probably due to the lack of proteolytic enzymes of the Mycobacterium, while organisms involved in PyS (*Staphylococcus aureus*, Enterobacter, and Salmonella) can produce hyaluronidase, resulting in intervertebral disc-lysis [43,55]. In some studies, disk space narrowing was similar in both types of spondylitis, possibly because a longer interval existed from presentation to MRI in the cases of TbS considered [49,50,51,52,53,54,55,56,57,58,59,60,61].

Another typical feature of TbS is a heterogeneous signal of the vertebral body both on T1w, on fluid-sensitive, and on CE sequences [49,50,56]. 

Thereby concluding, the main features of PyS are the involvement of the lumbar spine, poor and ill-defined enhancement of the paravertebral tissues, diffuse/homogeneous vertebral contrast enhancement of vertebral bodies, low degree destruction of the vertebral bodies, high and homogeneous signal intensity of the vertebral bodies on T2-weighted images, disc signal change, and disc height loss [43,49,50,51,52,53,54,55,56]. Additionally, anterior subligamentous spreading and posterior spine structures are generally not involved [60].

Interestingly, the SUV in TbS seems to be significantly higher than the SUV of other bacterial spondylodiscitis (on average 12.4 (range: 6–22) in patients with TbS, versus 7.3 (range: 4.1–13.4) in patients with PyS) [46].

A notable limitation of the above-mentioned differentiating imaging features among TbS and PyS is that these aspects are mainly qualitative (with the exception of SUV max and the number of vertebrae involved). This leads to possible diagnostic errors, especially for non-expert readers. Indeed, the current literature review aims to spread knowledge in this regard.

In Table 2, the main MRI differentiating features among TbS and PyS spondylodiscitis are summarized.

**Figure 6:**
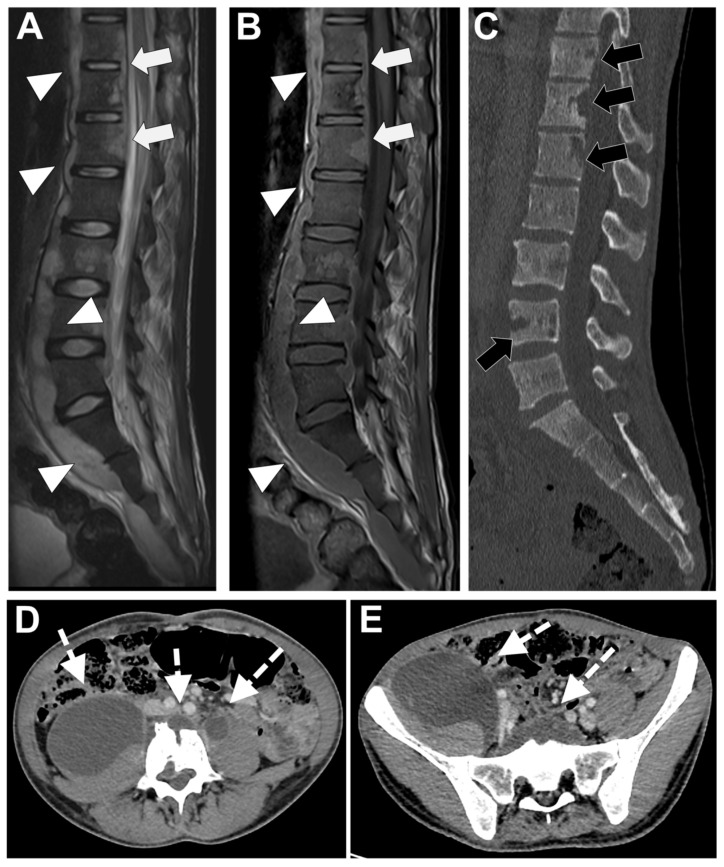
Tubercular spondylodiscitis. A 24-year-old man of Sudanese origin presented with thoracic and lumbar pain evolving for several months. An MRI was performed with (**A**) T2-weighted imaging (WI) and (**B**) contrast-enhanced (CE) T1-WI, as well as a CT-scan in bone kernel (**C**) and abdominal kernel after contrast medium injection (**D**,**E**). It demonstrates preserved disk but extensive sub ligamentous collections spreading along the anterior side of the thoracic and lumbar vertebral bodies (white arrowhead), but also along the posterior vertebral collateral ligament (white arrows) with large anterior and posterior erosions (black arrows). Please note the extensive collections spreading in the presacral space and along bilateral iliopsoas muscles without surrounding inflammation (white dashed arrows).

In Figure 6 an exemplificative case of TbS is presented.

**Figure 7:**
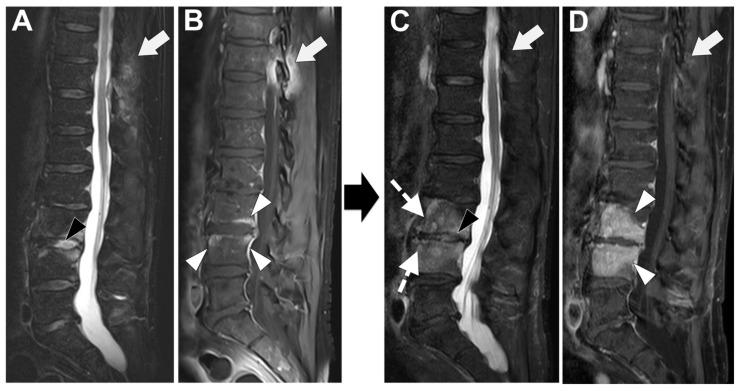
A 73-year-old male presented with a rapidly evolving lumbar pain and fever, with an inflammatory syndrome on blood samples. A first MRI was performed as infectious spondylodiscitis was suspected, which included (**A**) STIR T2-weighted imaging (WI) and (**B**) fat sat T1-WI after gadolinium chelates injection. It shows a high signal intensity (SI) of the L3-L4 disc while other disks are in lower signal (black arrowhead), as well as linear subchondral contrast enhancement (CE) of the L3-L4 endplates both linear and more pronounced at the upper anterior corner of the L4 vertebral body (white arrowheads). Moreover, the left T11-T12 facet joints displayed marked edema of the subchondral bone and surrounding tissues (white arrows). A control MRI with SITR T2-WI (**C**) and fat-suppressed CE-T1-WI (**D**) was performed one month later, demonstrating a marked narrowing of the L3-L4 disk (black arrowhead), erosions of the vertebral body (dashed white arrows), extensive edema in the L3 and L4 vertebral body (white arrowhead), a persisting arthritis involving the left T1-T12 facet joint. *Bacillus cereus* was found on the Bacterial analysis of the L3-L4 disk biopsy.

In Figure 7 an exemplificative case of PyS is presented.

### 3.2. Other Microorganisms

Other less frequently encountered microorganisms can demonstrate MRI features that may help guide the etiology of an infectious spondylodiscitis: 

Brucellosis. *Brucella* is a zoonose that belongs to gram-negative *coccobacilli*, which typically affects adult patients from South America, the Mediterranean basin, or the Middle East who can be exposed to unpasteurized infected milk or infected animals. Spondylodiscitis represents nearly half of the musculoskeletal involvement of Brucellosis (BrS) [58]. Knowing the MRI features of this type of spondylodiscitis can be helpful, as biopsies and blood cultures are often negative. Moreover, inflammatory syndrome (clinically and biologically) in BrS can be very scarce. The course of BrS is rather slow, with radiological abnormal findings usually appearing some weeks after the beginning of the disease. It is generally located in the lumbar spine, especially the anterosuperior corner, at a single level. The early involvement of the disk but not the posterior elements of the spine with a preserved vertebral body (despite large signal abnormalities) should raise attention [58]. It must be noted that peri-vertebral osseous construction, resembling anterior osteophytes, can occur [59].

Fungal spondylodiscitis. They are rare and are generally due to a hematogenous inoculation following a systemic infection in a deeply immunocompromised patient or with an intravenous drug addiction. The most frequent germs are *Candida albicans* and *Aspergillus fumigatus* or *flavius* [60]. The radiological features lack specificity. According to Simeone et al., partial disc involvement and focal soft-tissue abnormality (by opposition to diffuse involvement) may be more frequent in fungal spondylodisicitis compared to PyS [28,60].

### 3.3. Image-Guided Percutaneous Biopsy

An image-guided percutaneous biopsy is a safe and valid option to confirm the suspected diagnosis of spondylodiscitis and/or to achieve the exact microorganism involved.

**Figure 8:**
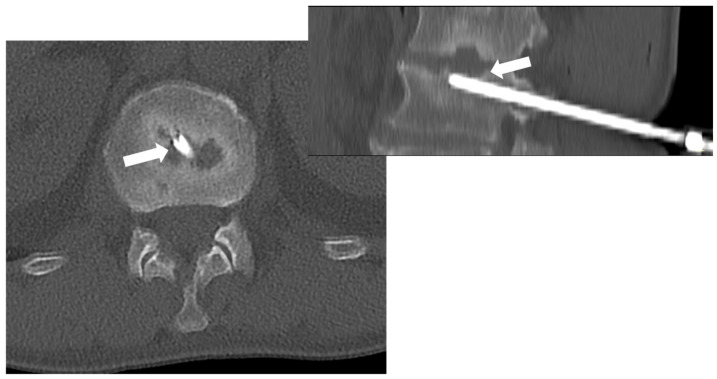
CT-guided biopsy in a 55-year-old male with suspected spondylodiscitis; CT (axial view on the left—sagittal reconstruction on the right) permits to guide the tip of the needle (8 gauge) into the end plate erosion (arrows) adjacent to the disc.

Among the imaging tools that can guide the biopsy procedure, CT is the most used and effective, particularly in reaching the spine safely. Indeed, CT guidance is superior to fluoroscopic guidance, especially for small spinal lesions. Most importantly, it can guide procedures in all skeletal areas (including spinal segments) (Figure 8) [67]. 

Ultrasound guidance is used in the spine only in selected cases, especially if a large paravertebral abscess is present [68].

CT-guided biopsy is effective in identifying an active bacterial infection of the spine, while its accuracy reduces significantly in chronic and/or inactive diseases as well as in fungal infections [69]. 

Chang et al. recently performed a systematic review and meta-analysis on image-guided biopsy for acute diskitis-osteomyelitis [70]. The article revealed that there were no statistically significant differences between image guidance (CT or Fluoroscopy) and diagnostic yield. The site where the biopsy samples were performed significantly influenced the microbiological diagnostic yield: 64.8% when performing the procedure on disc or paravertebral soft tissue involved and 45.5% on bone end plates (*p* < 0.001) [70].

Moreover, it is known that several factors are associated with the highest diagnostic yield on CT-guided biopsy for spondylodiscitis assessment and in general [67,71]; the main ones are summarized in Table 3.

## 4. Differential Diagnoses

Several diseases can mimic spinal infections radiologically. Again, radiologists can help avoid misdiagnoses thanks to CT and MRI features, interpreted in a specific clinical and biological context. Only the most classical differential diagnoses are detailed in this section.

### 4.1. Degenerative Endplates Changes

**Figure 9:**
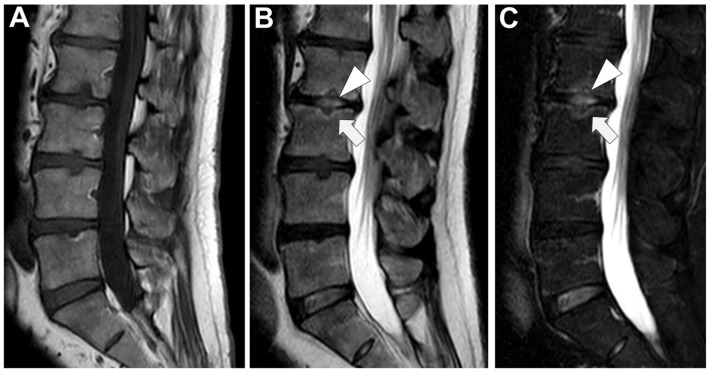
A 56-year-old male presented with a chronic and mechanic lumbar pain. An MRI was performed with (**A**) T1-weighted imaging (WI), (**B**) T2-WI, and (**C**) fat suppressed T2-WI. It demonstrates multiple Schmorl nodes (arrowheads) and a linear high signal intensity (SI) of the subchondral bones on both side of the L2-L3 level (arrows). There was no erosion, small anterior osteophyte, and degenerative disks. Hence, Modic 1 was diagnosed.

Degenerative end plate changes at the early inflammatory phase (Modic 1) can demonstrate some similar radiological features with infectious spondylodiscitis [72,73], that is to say, irregular end plate contours, possible subchondral cysts, and vertebral edema with a horizontal orientation (with high signal intensity on T2-WI—more pronounced with fat suppression method, low signal intensity on T1-WI, and, when performed, possible contrast-enhancement)—Figure 9. 

However, additional features can rectify a misdiagnosis. First, the disk thinning is generally extended to the whole disk with low disk signal intensity on T2-WI. Second, on the T1-WI sequence and DIXON fat-saturated sequence, there is still a fatty signal intensity of the end plate. Third, the end plate borders are usually spared and remain continuous. Fourth, the surrounding soft tissues and epidural spaces should be spared. Third, associations with Modic 2 (i.e., healing process with fatty replacement of the vertebral endplate) and Modic 3 (i.e., healing process with sclerosis or hardening of the vertebral end plate) changes are frequent. It must be noted that erosions can happen during this inflammatory degeneration of the disk but without major destruction. Lastly, it has been suggested that DWI could help discriminate Modic 1 from infectious spondylodiscitis, but heterogeneous acquisition parameters have precluded from identifying ADC cut-off with sufficient diagnostic accuracy [74]. However, some qualitative DWI characteristics seem to remain relevant to diagnose Modic 1, such as the ‘claw sign’ [75]. It consists of a linear and paired area with high signal intensity on DWI and well-defined margins, whereas infectious spondylodiscitis would provide ill-defined diffuse (or unpaired) signal abnormalities on DWI [74,75].

### 4.2. Andersson Lesion 

**Figure 10:**
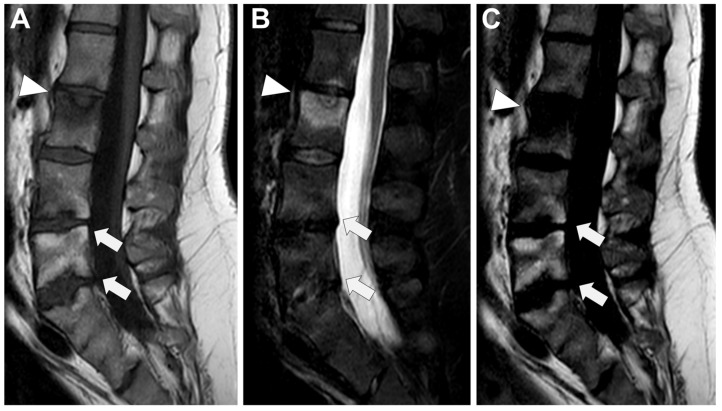
A 47-year-old male with a medical history of ankylosing spondylitis presented with the resurgence of upper lumbar pain with an inflammatory schedule. An MRI was performed, including (**A**) T1-weighted imaging (WI), (**B**) Dixon T2-WI with the Water Image (**B**), and the Fat image (**C**). This examination exhibits Andersson lesions of various ages. The white arrowhead shows the most recent lesions with a deep erosion in the middle of the upper L2 endplate with marked edema of the upper half of the L2 vertebral body. The white arrows show older lesions at the L4-L5 and L5-S1 levels with fatty replacement of the subchondral bone of the endplates.

The Andersson lesion is a relatively rare inflammatory disco-vertebral complication of ankylosing spondylitis, occurring in 1 to 28% of patients [76]. It could be due to a combination of acute inflammatory enthesopathy extending posteriorly to the disk and end plates and a microtraumatic process (Figure 10) [77].

The Andersson lesion is typically located at the thoracolumbar junction or lumbar spine and involves more than one level per patient. On CT and MRI, often central but also peripheral focal erosions are seen, with a varying depth, along with sclerosis of the end plates and marked bone edema on both sides of the disco-vertebral unit (with high signal intensity on fat-suppressed T2-WI and low signal intensity on T1-WI and possible contrast-enhancement if injection is performed). However, radiological features can help differentiate from infectious spondylodiscitis. First, there is no spreading to the paraspinal soft-tissue or to the epidural space. Second, other characteristics related to spondylarthritis are often observed such as inflammatory anterior enthesitis, fatty or sclerosing sequellae at the anterior corners of the vertebral body, syndesmophytosis, sacroiliitis, and inflammation of costovertebral and costotransverse joints [77].

### 4.3. Spinal Involvement in SAPHO Syndrome

The involvement of the axial skeleton in synovitis, acne, pustulosis, hyperostosis, and osteitis (SAPHO) syndrome occurs in 32–52% of patients. In this inflammatory disorder, the spinal lesions can occur from the mid-cervical spine to the sacrum but predominate on the anterior side of the thoracic or lumbar vertebral body [76]. A constant finding is an erosion of the vertebral corner, almost always at its anterior side [77,78,79]. Moreover, contiguous vertebral lesions are seen in 89% of patients, sometimes (about 17%) on each anterior side of the same disk, mimicking the early stage of PyS. Bone marrow edema within the vertebral body is classically found. Furthermore, prevertebral inflammatory tissue thickening (notably subligamentous anterior thickening) has been described in one-third of patients, as well as intradiscal abnormal high signal intensity on T2-WI, further resembling infectious spondylodiscitis [80]. However, the correct diagnosis of spinal SAPHO can be corrected. The involvement of other sites, such as sacroiliac and sternoclavicular joints, is frequent, as well as osteosclerosis of one or more levels, hyperostosis, or paravertebral ossification.

### 4.4. Micro-Crystalline Spondylodiscitis

**Figure 11:**
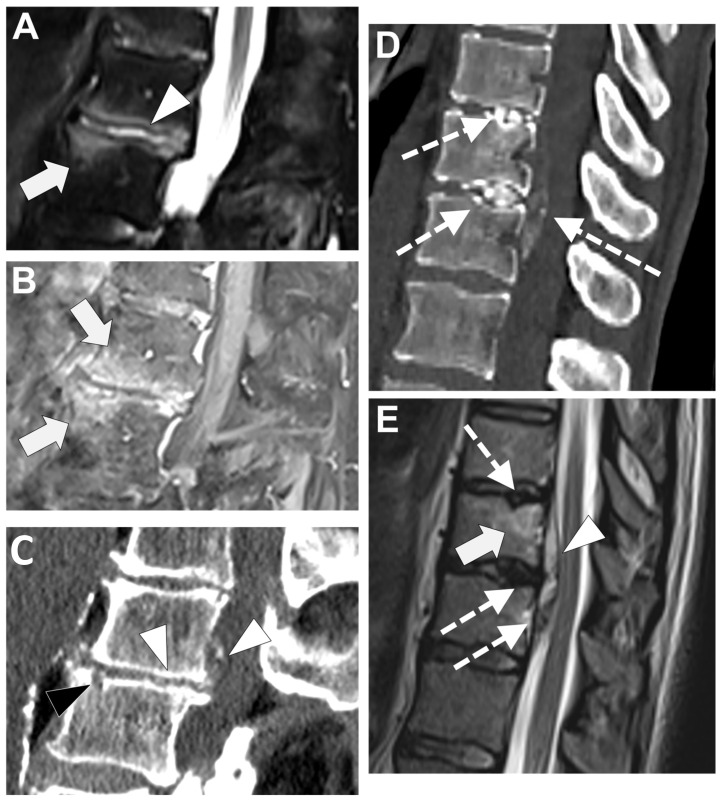
Examples of microcristalline spinal disorders. A 78-year-old male presented with acute inflammatory pain of the upper lumbar spine. An MRI was performed with (**A**) STIR T2-weighted imaging (WI) and (**B**) fat sat T1-WI after gadolinium chelates injection, followed by a CT scan (**C**). It shows a linear high signal (SI) of a narrowed disk (white arrowhead) on STIR T2-WI with multiple small powdery calcifications on CT-scan, typical of chondrocalcinosis. It was associated with subchondral edema (white arrows) and a small endplate erosion (black arrowhead). A 35-year-old woman presented to the emergency for acute and intense dorsal pain. On CT-scan (**D**), gross and dense calcifications of two adjacent disks were observed (typical of hydroxyapatite) with a migration in the anterior epidural space (dashed white arrow). On T2-WI (**E**), the low T2-SI of the calcification can be observed (dashed white arrows), as well as a subtle edema of the posterior vertebral corner (white arrow) and the thickening of the epidural space (white arrowhead).

All crystal diseases can lead to intra-disk deposits that are generally asymptomatic but can provide acute inflammatory pain mimicking infectious spondylodiscitis. Any level can be affected but with some specific topography depending on the subtype of the crystal deposit. Hence, in adults, hydroxyapatite deposition predominates in the centrum of the disk at the thoracic level, while calcium pyrophosphate crystal deposition is more peripheral, powdery, and multiple within the intervertebral disc (with, often, multiple levels involved, as well as the posterior element of the spine) [73,81]. Vertebral body edema can be observed, as well as surrounding tissues’ inflammatory reaction and intra-discal abnormal signal intensities (due to the crystal—with low signal intensity on T1-WI and T2-WI—and to the inflammation—with high signal intensity on fat-suppressed T2-WI). A CT scan is crucial to rectify the diagnosis, showing the spontaneous high densities of the crystal (Figure 11) [73,81].

### 4.5. Destructive Spondyloarthropathy in Hemodialyzed Patients

This non-infectious spondyloarthropathy belongs to the spectrum of renal osteodystrophy and is due to the deposition of amyloidosis in the disk and in the ligamentum flavum. Its presence is correlated with the duration of hemodialysis and with higher levels of beta2-microglobulin, parathyroid hormones, and alkaline phosphatase [80,81]. It usually involves the lower cervical spine and the cervico-thoracic junction, and both the disk and the posterior elements of the spine. On radiographs, CT, and MRI, this disease of immunocompromised patients can resemble infectious spondylodiscitis as it typically demonstrates a marked narrowing of the intervertebral disk space, subchondral erosion and cysts in the end plates, and end plate edema with contrast enhancement on fat suppressed CE-T1-WI [82,83]. 

### 4.6. Neuropathic Spinal Arthropathy

**Figure 12:**
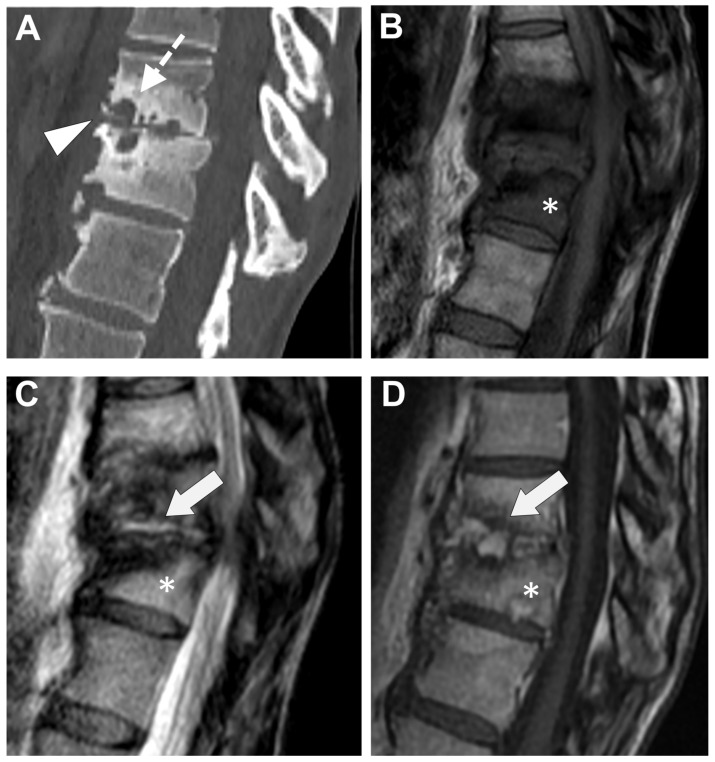
A 67-year-old woman with a medical history of cervical spinal cord injury several years before presented with a rapidly evolving spine deformation at the thoracolumbar junction (kyphosis). On CT (**A**), there was a strong narrowing of the disc (white arrowhead) with several erosions of endplates from each side (white dashed arrows) and osteosclerosis of the vertebral body. An MRI was performed with (**B**) T1-weighted imaging (WI), (**C**) T2-WI, and (**D**) T1-WI after gadolinium chelates injection. This examination demonstrates abnormal signal intensities (SIs) of the disk with SIs (fluid-like) on T2-WI, with a contrast enhancement (white arrows). The vertebral bodies showed an edema with low SIs on T1-WI, high SI on T2-WI, and contrast enhancement (asterisk).

Also named ‘Charcot’s spine,’ this is a rare complication of chronic neuropathic disorders such as diabetes mellitus, spinal cord injury, or syphilis. It corresponds to progressive degeneration of the disco-vertebral and facet joints secondary to the loss of proprioception, leading to abnormal stress on the spine (especially at the thoracolumbar and lumbosacral junctions) [84].—Figure 12. 

Thus, bone resorption, erosion, and destruction of the discs and end plates with bone marrow edema (possibly spreading to the surrounding soft tissues and showing contrast enhancement) can mimic infectious spondylitis. However, the following characteristics can help make the correct diagnosis. First, there is often still a vacuum disk phenomenon in the Charcot spine. Osseous debris due to bone fragmentations is frequently observed and is associated with bone sclerosis. Lastly, this disease results in joint dislocation of the facet and disco-vertebral joints, which become abnormally mobile, resulting in spine deformation [85]. Yet, it must be noted that infections of the Charcot’s spine are not exceptional and have been reported in 17% of cases [85].

## 5. Future Perspective

Since ^18^F-FDG-PET/CT provides sensitivity and a negative predictive value, when CT scans and CE MRIs of good quality (including the T1-WI, T2-WI, DWI, Dixon sequence, and fat-suppressed CE-T1-DWI) provide diagnostic accuracy plus specificity and discriminant features against differential diagnoses, one can expect potentiation of their added value, even though no studies have ever compared CE MRI, ^18^F-FDG-PET/CT, and PET/MRI in the setting of suspected infectious spondylitis [86].

Radiomics is a relatively recent field of research that consists of (i) extracting a large number of numeric variables quantifying the texture and the shape of ‘objects of interest’ on imaging (named the radiomics features, usually hundreds) and (ii) of training machine-learning algorithms in order to make predictions based on these radiomics features [87,88]. This approach requires standardized acquisitions, post-processing pipelines, and a well-explained statistical learning pipeline to ensure their reproducibility across centers [87,88]. Radiomics have been successfully applied in oncologic imaging, for instance, to differentiate between malignant and benign tumors, to identify molecular subtypes of cancers, or to predict the response to treatment and the patients’ survivals. Recently, these quantitative tools, with the aid of deep learning algorithms, machine learning, and radiomics analyses, have also been applied to spinal degenerative diseases [89,90,91]. Nonetheless, in a few recent articles, these new tools have also been used with the goal of a specific diagnosis. In 2018, Kim et al. used a deep convolutional neural network-based MRI algorithm to differentiate between tuberculous and pyogenic spondylodiscitis [92]. In 2024, Yasin et al. effectively performed an MRI-based radiomics analysis to differentiate *Brucella* and pyogenic spondylodiscitis [93].

Regarding infectious spondylitis, to the best of our knowledge, no studies have ever tested radiomics alone or combined with classical radiological features, although it could help to address various issues, notably the differentiation of TbS from PyS, or infectious spondylitis from Modic 1 or other non-infectious disco-vertebral inflammatory disorders. 

## 6. Conclusions

To conclude, this exhaustive review provides a comprehensive overview of the imaging features of infectious spondylodiscitis. General radiologists and radiologists with expertise in musculoskeletal imaging should master the characteristics of this disease on CT and MRI as its incidence should continue to increase. In particular, the early and subtle imaging features of infectious spondylodiscitis must be recognized to avoid diagnostic delays and rapidly providing the adequate treatment to patients. Moreover, both CE MRIs and CTs can help identifying the underlying microorganisms and differentiate the most frequent subtypes, i.e., mycobacterium tuberculosis and pyogenic bacteria, which can be valuable when hemoculture and invasive samples are non-contributive. Furthermore, imaging can correct misdiagnosis of infectious spondylitis. Indeed, while clinical and biological features can overlap between degenerative, inflammatory and infectious spondylodiscitis, CT and MRI can provide additional features to perform an accurate diagnosis and to avoid inappropriate percutaneous biopsy and anti-bacterium treatments. Finally, the imaging of infectious spondylodiscitis could benefit from the potentiation of ^18^F-FDG-PET/CT and MRI and from a radiomics approach to enhance its current performances.

## Figures and Tables

**Table 1:** Summary of the diagnostic characteristics of the main imaging tools for spondylodiscitis diagnosis.

Conventional Radiography	Computed Tomography	Magnetic Resonance Imaging	PET-CT
Low sensitivity and specificity Firs imaging tool Fast acquisition time Inexpensive Scarce evaluation of soft tissue and neural structures	High sensitivity in the detection of endplate erosions and bone disruption Fast acquisition time Possible guidance for biopsy Good evaluation of soft tissue with the use of intravenous contrast media injection Scarce evaluation of neural structures	Preferred imaging method with very high sensitivity and specificity Long acquisition time Optimal evaluation of soft tissue even without the use of intravenous contrast media injection Optimal evaluation of neural structures Relatively expensive Help in differentiating Pyogenic vs. Tubercular infections (or different microorganisms involved)	High sensitivity Relatively fast acquisition time Scarce evaluation of neural structures Good evaluation of soft tissue Relatively expensive Help in differentiating Pyogenic vs. Tubercular infections (or different microorganisms involved)

**Table 2:** Imaging features in the differential diagnosis between TbS and PyS.

Imaging Features	Tuberculous Spondylitis (TbS)	Pyogenic Spondylitis (PyS)
Thoracic spine involvement	Present	Usually absent
Subligamentous spread to 3 or more vertebral bodies	Multiple body involvement	Usual involvement ≤ 2 vertebral bodies
Involvement of posterior elements	Present	Usually absent
(MRI) Paraspinal signal	Well-defined	Ill-defined
Paraspinal abscess	75% of cases	39–40% of cases
Epidural abscess	56–60% of cases	11–15% of cases
Intraosseous abscess	Present	Absent
Abscess wall	Thin and smooth	Thick and irregular
(MRI) Vertebral enhancement	Focal/heterogeneous	Diffuse/homogeneous
(MRI) Vertebral signal in T2 images	Heterogeneous	Hyperintense/homogeneous
(MRI) Vertebral signal in T1 images	Heterogeneous	Hypointense/homogeneous
Destruction of vertebral bodies	Frequent and more severe	Infrequent and mild to moderate
Disc destruction	Mild to moderate	Severe to complete
(PET) FDG SUV	Higher (mean = 12)	Lower (mean = 7)

**Table 3:** Factors associated with higher or lower diagnostic yield on CT-guided biopsies.

CT-Guided Biopsy for Spondylodiscitis— Factors Associated with Diagnostic Yield	
Lower Diagnostic Rate	Higher Diagnostic Rate
Small lesion size	Large lesion size
Single bone sample	Multiple bone samples
Short sample (short needle penetration in the lesion/perpendicular needle trajectory)	Large sample (long needle penetration in the lesion/oblique needle trajectory)
Targeting the vertebral bone or endplates only	Targeting the disc, and/or soft-tissue involvement, and/or Fluid collection aspiration.
Target lesion not visible on CT	Target lesion visible on CT
Fungal Infection	Mycobacterium Tubercolosis

## Data Availability

Data are contained within the article.

## References

[1] Grammatico L., Baron S., Rusch E., Lepage B., Surer N., Desenclos J.C., Besnier J.M. (2008). Epidemiology of vertebral osteomyelitis (VO) in France: Analysis of hospital-discharge data 2002–2003. Epidemiol. Infect..

[2] Chelsom J., Solberg C.O. (1998). Vertebral osteomyelitis at a Norwegian university hospital 1987–1997: Clinical features, laboratory findings and outcome. Scand. J. Infect. Dis..

[3] Joughin E., McDougall C., Parfitt C., Yong-Hing K., Kirkaldy-Willis W.H. (1991). Causes and clinical management of vertebral osteomyelitis in Saskatchewan. Spine.

[4] Fantoni M., Trecarichi E.M., Rossi B., Mazzotta V., Di Giacomo G., Nasto L.A., Di Meco E., Pola E. (2012). Epidemiological and clinical features of pyogenic spondylodiscitis. Eur. Rev. Med. Pharmacol. Sci..

[5] Son H.J., Kim M., Kim D.H., Kang C.N. (2023). Incidence and treatment trends of infectious spondylodiscitis in South Korea: A nationwide population-based study. PLoS ONE.

[6] Govender S. (2005). Spinal infections. J. Bone Joint Surg Br..

[7] Nasto L.A., Colangelo D., Rossi B., Fantoni M., Pola E. (2012). Post-operative spondylodiscitis. Eur. Rev. Med. Pharmacol. Sci..

[8] Lang S., Walter N., Schindler M., Baertl S., Szymski D., Loibl M., Alt V., Rupp M. (2023). The Epidemiology of Spondylodiscitis in Germany: A Descriptive Report of Incidence Rates, Pathogens, In-Hospital Mortality, and Hospital Stays between 2010 and 2020. J. Clin. Med..

[9] Kramer A., Thavarajasingam S.G., Neuhoff J., Ponniah H.S., Ramsay D.S.C., Demetriades A.K., Davies B.M., Shiban E., Ringel F. (2023). Epidemiological trends of pyogenic spondylodiscitis in Germany: An EANS Spine Section Study. Sci. Rep..

[10] Pigrau C., Almirante B., Flores X., Falco V., Rodríguez D., Gasser I., Villanueva C., Pahissa A. (2005). Spontaneous pyogenic vertebral osteomyelitis and endocarditis: Incidence, risk factors, and outcome. Am. J. Med..

[11] Jimenez-Mejias M.E., de Dios Colmenero J., Sanchez-Lora F.J., Palomino-Nicás J., Reguera J.M., de la Heras J.G., García-Ordonez M.A., Pachon J. (1999). Postoperative spondylodiskitis: Etiology, clinical findings, prognosis, and comparison with nonoperative pyogenic spondylodiskitis. Clin. Infect. Dis..

[12] Gentile L., Benazzo F., De Rosa F., Boriani S., Dallagiacoma G., Franceschetti G., Gaeta M., Cuzzocrea F. (2019). A systematic review: Characteristics, complications and treatment of spondylodiscitis. Eur. Rev. Med. Pharmacol. Sci..

[13] Sato K., Yamada K., Yokosuka K., Yoshida T., Goto M., Matsubara T., Iwahashi S., Shimazaki T., Nagata K., Shiba N. (2019). Pyogenic Spondylitis: Clinical Features, Diagnosis and Treatment. Kurume Med. J..

[14] Asperges E., Albi G., Truffelli F., Salvaderi A., Puci F., Sangani A., Zuccaro V., Scotti V., Orsolini P., Brunetti E. (2023). Fungal Osteomyelitis: A Systematic Review of Reported Cases. Microorganisms.

[15] Koutserimpas C., Chamakioti I., Naoum S., Raptis K., Alpantaki K., Kofteridis D.P., Samonis G. (2021). Spondylodiscitis Caused by Aspergillus Species. Diagnostics.

[16] Petkova A.S., Zhelyazkov C.B., Kitov B.D. (2017). Spontaneous Spondylodiscitis—Epidemiology, Clinical Features, Diagnosis and Treatment. Folia Med..

[17] Pola E., Taccari F., Autore G., Giovannenze F., Pambianco V., Cauda R., Maccauro G., Fantoni M. (2018). Multidisciplinary management of pyogenic spondylodiscitis: Epidemiological and clinical features, prognostic factors and long-term outcomes in 207 patients. Eur. Spine J..

[18] Shanmuganathan R., Ramachandran K., Shetty A.P., Kanna R.M. (2023). Active tuberculosis of spine: Current updates. N. Am. Spine Soc. J..

[19] Hopkinson N., Stevenson J., Benjamin S. (2001). A case ascertainment study of septic discitis: Clinical, microbiological and radiological features. QJM.

[20] Cottle L., Riordan T. (2008). Infectious spondylodiscitis. J. Infect..

[21] Acosta F.L., Galvez L.F., Aryan H.E., Ames C.P. (2006). Recent advances: Infections of the spine. Curr. Infect. Dis. Rep..

[22] Butler J.S., Shelly M.J., Timlin M., Powderly W.G., O’Byrne J.M. (2006). Nontuberculous pyogenic spinal infection in adults: A 12-year experience from a tertiary referral center. Spine.

[23] Nasto L.A., Fantoni M., Cipolloni V., Piccone L., Pola E., Schiavone Panni A. (2021). A Detailed Analysis of Clinical Features and Outcomes of Patients with Pyogenic Spondylodiscitis Presenting without Axial Back Pain. Trop. Med. Infect. Dis..

[24] Berbari E.F., Kanj S.S., Kowalski T.J., Darouiche R.O., Widmer A.F., Schmitt S.K., Hendershot E.F., Holtom P.D., Huddleston P.M., Petermann G.W. (2015). 2015 Infectious Diseases Society of America (IDSA) Clinical Practice Guidelines for the Diagnosis and Treatment of Native Vertebral Osteomyelitis in Adults. Clin. Infect. Dis..

[25] Mylona E., Samarkos M., Kakalou E., Fanourgiakis P., Skoutelis A. (2009). Pyogenic vertebral osteomyelitis: A systematic review of clinical characteristics. Semin. Arthritis Rheum..

[26] Jensen A.G., Espersen F., Skinhoj P., Frimodt-Moller N. (1998). Bacteremic *Staphylococcus aureus* spondylitis. Arch. Intern. Med..

[27] Lertudomphonwanit T., Somboonprasert C., Lilakhunakon K., Jaovisidha S., Ruangchaijatuporn T., Fuangfa P., Rattanasiri S., Watcharananan S., Chanplakorn P. (2023). A clinical prediction model to differentiate tuberculous spondylodiscitis from pyogenic spontaneous spondylodiscitis. PLoS ONE.

[28] Simeone F.J., Husseini J.S., Yeh K.J., Lozano-Calderon S., Nelson S.B., Chang C.Y. (2020). MRI and clinical features of acute fungal discitis/osteomyelitis. Eur. Radiol..

[29] An H.S., Seldomridge J.A. (2006). Spinal infections: Diagnostic tests and imaging studies. Clin. Orthop. Relat. Res..

[30] Go J.L., Rothman S., Prosper A., Silbergleit R., Lerner A. (2012). Spine infections. Neuroimaging Clin. N. Am..

[31] Stäbler A., Reiser M.F. (2001). Imaging of spinal infection. Radiol. Clin. N. Am..

[32] Grados F., Lescure F.X., Senneville E., Flipo R.M., Schmit J.L., Fardellone P. (2007). Suggestions for managing pyogenic (non-tuberculous) discitis in adults. Joint Bone Spine.

[33] Arbelaez A., Restrepo F., Castillo M. (2014). Spinal infections: Clinical and imaging features. Top. Magn. Reason. Imaging.

[34] Heyde C.E., Spiegl U.J.A., Voelker A., von der Hoeh N., Henkelmann J. (2023). Imaging in the Diagnosis of Nonspecific pyogenic Spondylodiskitis. J. Neurol. Surg. A Cent. Eur. Neurosurg..

[35] Brant-Zawadzki M., Burke V.D., Jeffrey R.B. (1983). CT in the evaluation of spine infection. Spine.

[36] McGahan J.P., Dublin A.B. (1985). Evaluation of spinal infections by plain radiographs, computed tomography, intrathecal metrizamide, and CT-guided biopsy. Diagn. Imaging Clin. Med..

[37] Raghavan M., Lazzeri E., Palestro C.J. (2018). Imaging of Spondylodiscitis. Semin. Nucl. Med..

[38] Dagirmanjian A., Schils J., McHenry M., Modic M.T. (1996). MR imaging of vertebral osteomyelitis revisited. AJR Am. J. Roentgenol..

[39] Ledermann H.P., Schweitzer M.E., Morrison W.B., Carrino J.A. (2003). MR imaging findings in spinal infections: Rules or myths?. Radiology.

[40] Longo M., Granata F., Ricciardi K., Gaeta M., Blandino A. (2003). Contrast-enhanced MR imaging with fat suppression in adult-onset septic spondylodiscitis. Eur. Radiol..

[41] Diehn F.E. (2012). Imaging of spine infection. Radiol. Clin. N. Am..

[42] Tsantes A.G., Papadopoulos D.V., Vrioni G., Sioutis S., Sapkas G., Benzakour A., Benzakour T., Angelini A., Ruggieri P., Mavrogenis A.F. (2020). Spinal Infections: An Update. Microorganisms.

[43] Salaffi F., Ceccarelli L., Carotti M., Di Carlo M., Polonara G., Facchini G., Golfieri R., Giovagnoni A. (2021). Differentiation between infectious spondylodiscitis versus inflammatory or degenerative spinal changes: How can magnetic resonance imaging help the clinician?. Radiol. Med..

[44] Righi E., Carnelutti A., Muser D., Di Gregorio F., Cadeo B., Melchioretto G., Merelli M., Alavi A., Bassetti M. (2020). Incremental value of FDG-PET/CT to monitor treatment response in infectious spondylodiscitis. Skeletal Radiol..

[45] Fuster D., Solà O., Soriano A., Monegal A., Setoain X., Tomás X., Garcia S., Mensa J., Rubello D., Pons F. (2012). A prospective study comparing whole-body FDG PET/CT to combined planar bone scan with 67Ga SPECT/CT in the Diagnosis of Spondylodiskitis. Clin. Nucl. Med..

[46] Bassetti M., Merelli M., Di Gregorio F., Della Siega P., Screm M., Scarparo C., Righi E. (2017). Higher fluorine-18 fluorodeoxyglucose positron emission tomography (FDG-PET) uptake in tuberculous compared to bacterial spondylodiscitis. Skeletal Radiol..

[47] Martinez V., Castilla-Lievre M.A., Guillet-Caruba C., Grenier G., Fior R., Desarnaud S., Doucet-Populaire F., Boué F. (2012). (18)F-FDG PET/CT in tuberculosis: An early non-invasive marker of therapeutic response. Int. J. Tuberc. Lung Dis..

[48] Love C., Patel M., Lonner B.S., Tomas M.B., Palestro C.J. (2000). Diagnosing spinal osteomyelitis: A comparison of bone and Ga-67 scintigraphy and magnetic resonance imaging. Clin. Nucl. Med..

[49] Kayani I., Syed I., Saifuddin A., Green R., MacSweeney F. (2004). Vertebral osteomyelitis without disc involvement. Clin. Radiol..

[50] Tali E.T., Oner A.Y., Koc A.M. (2015). Pyogenic spinal infections. Neuroimaging Clin. N. Am..

[51] Babic M., Ilaslan H., Shrestha N., Simpfendorfer C.S. (2020). Isolated septic facet joints: An underdiagnosed distinct clinical entity. Skeletal Radiol..

[52] Zhang N., Zeng X., He L., Liu Z., Liu J., Zhang Z., Chen X., Shu Y. (2019). The Value of MR Imaging in Comparative Analysis of Spinal Infection in Adults: Pyogenic Versus Tuberculous. World Neurosurg..

[53] Kumar Y., Gupta N., Chhabra A., Fukuda T., Soni N., Hayashi D. (2017). Magnetic resonance imaging of bacterial and tuberculous spondylodiscitis with associated complications and non-infectious spinal pathology mimicking infections: A pictorial review. BMC Musculoskelet. Disord..

[54] Henkelmann J., Bremicker K., Denecke T., Hoffmann K.-T., Henkelmann R., Heyde C.-E., Sabri O., Purz S. (2021). Clinical suspicion of spondylodiscitis with equivocal MRI findings: Does diffusion-weighted imaging prove helpful here?. Acta Radiol..

[55] Naselli N., Facchini G., Lima G.M., Evangelisti G., Ponti F., Miceli M., Spinnato P. (2022). MRI in differential diagnosis between tuberculous and pyogenic spondylodiscitis. Eur. Spine J..

[56] Galhotra R.D., Jain T., Sandhu P., Galhotra V. (2015). Utility of magnetic resonance imaging in the differential diagnosis of tubercular and pyogenic spondylodiscitis. J. Nat. Sci. Biol. Med..

[57] Prodi E., Grassi R., Iacobellis F., Cianfoni A. (2016). Imaging in Spondylodiskitis. Magn. Reason. Imaging Clin. N. Am..

[58] al-Shahed M.S., Sharif H.S., Haddad M.C., Aabed M.Y., Sammak B.M., Mutairi M.A. (1994). Imaging features of musculoskeletal brucellosis. Radiographics.

[59] Chelli Bouaziz M., Ladeb M.F., Chakroun M., Chaabane S. (2008). Spinal brucellosis: A review. Skeletal Radiol..

[60] Williams R.L., Fukui M.B., Meltzer C.C., Swarnkar A., Johnson D.W., Welch W. (1999). Fungal spinal osteomyelitis in the immunocompromised patient: MR findings in three cases. AJNR Am. J. Neuroradiol..

[61] Jung N.Y., Jee W.H., Ha K.Y., Park C.K., Byun J.Y. (2004). Discrimination of tuberculous spondylitis from pyogenic spondylitis on MRI. AJR Am. J. Roentgenol..

[62] Lee K.Y. (2014). Comparison of pyogenic spondylitis and tuberculous spondylitis. Asian Spine J..

[63] Ling-Shan C., Zheng-Qiu Z., Jing L., Rui Z., Li-Fang L., Zhi-Tao W., Zhong-Qiu W. (2023). Magnetic resonance imaging features for differentiating tuberculous from pyogenic spondylitis: A meta-analysis. Skeletal Radiol..

[64] Wang J., Li Z., Chi X., Chen Y., Wang H., Wang X., Cui K., Wang Q., Lu T., Zheng J.M. (2024). Development of a Diagnostic Model for Differentiating Tuberculous Spondylitis and Pyogenic Spondylitis With MRI: A Multicenter Retrospective Observational Study. Spine.

[65] Gupta N., Kadavigere R., Malla S., Bhat S.N., Saravu K. (2023). Differentiating tubercular from pyogenic causes of spine involvement on Magnetic Resonance Imaging. Infez. Med..

[66] Frel M., Białecki J., Wieczorek J., Paluch Ł., Dąbrowska-Thing A., Walecki J. (2017). Magnetic Resonance Imaging in Differentatial Diagnosis of Pyogenic Spondylodiscitis and Tuberculous Spondylodiscitis. Pol. J. Radiol..

[67] Spinnato P., Colangeli M., Rinaldi R., Ponti F. (2023). Percutaneous CT-Guided Bone Biopsies: Indications, Feasibility and Diagnostic Yield in the Different Skeletal Sites-From the Skull to the Toe. Diagnostics.

[68] Ponti F., Arioli A., Longo C., Miceli M., Colangeli M., Papalexis N., Spinnato P. (2023). Ultrasound-Guided Percutaneous Bone Biopsy: Feasibility, Diagnostic Yield and Technical Notes. Diagnostics.

[69] Chew F.S., Kline M.J. (2001). Diagnostic yield of CT-guided percutaneous aspiration procedures in suspected spontaneous infectious diskitis. Radiology.

[70] Chang C.Y., Pelzl C., Jesse M.K., Habibollahi S., Habib U., Gyftopoulos S. (2023). Image-Guided Biopsy in Acute Diskitis-Osteomyelitis: A Systematic Review and Meta-Analysis. AJR Am. J. Roentgenol..

[71] Li Y., Du Y., Luo T.Y., Yang H.F., Yu J.H., Xu X.X., Zheng H.J., Li B. (2014). Factors influencing diagnostic yield of CT-guided percutaneous core needle biopsy for bone lesions. Clin. Radiol..

[72] Zhang C., Liu S. (2023). The advancement of MRI in differentiating Modic type I degenerative changes from early spinal infections. Br. J. Radiol..

[73] Boudabbous S., Paulin E.N., Delattre B.M.A., Hamard M., Vargas M.I. (2021). Spinal disorders mimicking infection. Insights Imaging.

[74] Daghighi M.H., Poureisa M., Safarpour M., Behzadmehr R., Fouladi D.F., Meshkini A., Varshochi M., Kiani Nazarlou A. (2016). Diffusion-weighted magnetic resonance imaging in differentiating acute infectious spondylitis from degenerative Modic type 1 change; the role of b-value, apparent diffusion coefficient, claw sign and amorphous increased signal. Br. J. Radiol..

[75] Patel K.B., Poplawski M.M., Pawha P.S., Naidich T.P., Tanenbaum L.N. (2014). Diffusion-weighted MRI “claw sign” improves differentiation of infectious from degenerative modic type 1 signal changes of the spine. AJNR Am. J. Neuroradiol..

[76] Bron J.L., de Vries M.K., Snieders M.N., van der Horst-Bruinsma I.E., van Royen B.J. (2009). Discovertebral (Andersson) lesions of the spine in ankylosing spondylitis revisited. Clin. Rheumatol..

[77] Park Y.S., Kim J.H., Ryu J.A., Kim T.H. (2011). The Andersson lesion in ankylosing spondylitis: Distinguishing between the inflammatory and traumatic subtypes. J. Bone Joint Surg. Br..

[78] Leone A., Cassar-Pullicino V.N., Casale R., Magarelli N., Semprini A., Colosimo C. (2015). The SAPHO syndrome revisited with an emphasis on spinal manifestations. Skeletal Radiol..

[79] Laredo J.D., Vuillemin-Bodaghi V., Boutry N., Cotten A., Parlier-Cuau C. (2007). SAPHO syndrome: MR appearance of vertebral involvement. Radiology.

[80] McGauvran A.M., Kotsenas A.L., Diehn F.E., Wald J.T., Carr C.M., Morris J.M. (2016). SAPHO Syndrome: Imaging Findings of Vertebral Involvement. AJNR Am. J. Neuroradiol..

[81] Shah A., Botchu R., Grainger M.F., Davies A.M., James S.L. (2015). Acute symptomatic calcific discitis in adults: A case report and review of literature. Skeletal Radiol..

[82] Maruyama H., Gejyo F., Arakawa M. (1992). Clinical studies of destructive spondyloarthropathy in long-term hemodialysis patients. Nephron.

[83] Leone A., Sundaram M., Cerase A., Magnavita N., Tazza L., Marano P. (2001). Destructive spondyloarthropathy of the cervical spine in long-term hemodialyzed patients: A five-year clinical radiological prospective study. Skeletal Radiol..

[84] Ledbetter L.N., Salzman K.L., Sanders R.K., Shah L.M. (2016). Spinal Neuroarthropathy: Pathophysiology, Clinical and Imaging Features, and Differential Diagnosis. Radiographics.

[85] Wagner S.C., Schweitzer M.E., Morrison W.B., Przybylski G.J., Parker L. (2000). Can imaging findings help differentiate spinal neuropathic arthropathy from disk space infection? Initial experience. Radiology.

[86] Telli T., Desaulniers M., Pyka T., Caobelli F., Forstmann S., Umutlu L., Fendler W.P., Rominger A., Herrmann K., Seifert R. (2023). What Role Does PET/MRI Play in Musculoskeletal Disorders?. Semin. Nucl. Med..

[87] Lambin P., Leijenaar R.T.H., Deist T.M., Peerlings J., de Jong E.E.C., van Timmeren J., Sanduleanu S., Larue R.T.H.M., Even A.J.G., Jochems A. (2017). Radiomics: The bridge between medical imaging and personalized medicine. Nat. Rev. Clin. Oncol..

[88] Limkin E.J., Sun R., Dercle L., Zacharaki E.I., Robert C., Reuzé S., Schernberg A., Paragios N., Deutsch E., Ferté C. (2017). Promises and challenges for the implementation of computational medical imaging (radiomics) in oncology. Ann. Oncol..

[89] Jiang C., Zhang J., Li W., Li Y., Ni M., Jin D., Zhang Y., Jiang L., Yuan H. (2024). Deep Learning Imaging Reconstruction Algorithm for Carotid Dual Energy CT Angiography: Opportunistic Evaluation of Cervical Intervertebral Discs—A Preliminary Study. J. Imaging Inform. Med..

[90] Staartjes V.E., Quddusi A., Klukowska A.M., Schröder M.L. (2020). Initial classification of low back and leg pain based on objective functional testing: A pilot study of machine learning applied to diagnostics. Eur. Spine J..

[91] Grob A., Loibl M., Jamaludin A., Winklhofer S., Fairbank J.C.T., Fekete T., Porchet F., Mannion A.F. (2022). External validation of the deep learning system “SpineNet” for grading radiological features of degeneration on MRIs of the lumbar spine. Eur. Spine J..

[92] Kim K., Kim S., Lee Y.H., Lee S.H., Lee H.S., Kim S. (2018). Performance of the deep convolutional neural network based magnetic resonance image scoring algorithm for differentiating between tuberculous and pyogenic spondylitis. Sci. Rep..

[93] Yasin P., Yimit Y., Abliz D., Mardan M., Xu T., Yusufu A., Cai X., Sheng W., Mamat M. (2023). MRI-based interpretable radiomics nomogram for discrimination between Brucella spondylitis and Pyogenic spondylitis. Heliyon.

